# Supplementary material for: Assessment of Knowledge, Practice and Guidelines towards the Novel COVID-19 among Eye Care Practitioners in Nigeria–A Survey-Based Study
Source: Int J Environ Res Public Health. 2020 Jul 16;17(14):5141. doi: 10.3390/ijerph17145141 (PMC7399827; doi:10.3390/ijerph17145141)
Supplement: Supplementary file 1 [file ijerph-17-05141-s001.pdf]

International Journal of
Environmental Research and Public Health

MDPI

Table S1: Survey tool used in this study

Assessment of knowledge, practice and guidance on covid-19 among eye care practitioners in Nigeria- survey based study

I consent to voluntarily contribute to the knowledge and practice on Coronavirus disease (COVID-19) Pandemic, and I agree to participate in this study

- Yes
- No

SECTION A- Demographic characteristics

1. What is your gender?
   a. Female
   b. Male
   c. Others (please specify): ________
2. In which State in Nigeria do you practice? __________________________ .
3. What is your age? __________________________.
4. Marital status
   a. Married
   b. Single
   c. Divorced/Separated
   d. Widow/Widower
5. Religion
   a. Christian
   b. Muslim
   c. African traditional
   d. Other (please specify): _________________
6. Highest educational qualification
   a. University degree (Bachelors/Doctor of Optometry/Professional degree)
   b. Fellowship
   c. Post graduate degree (Masters)
   d. Doctor of Philosophy (PhD)
   e. Others (please specify): _____________
7. What is your job title?
   a. Ophthalmologist
   b. Optometrist
   c. Ophthalmic nurse
   d. Optician
   e. Others (please specify): _____________
8. Where do you work?
   a. Public Hospital
   b. Private Hospital/Clinic
   c. Other (please specify): _____________
9. What is your employment status?
   a. Self employed
   b. Government employee

c. Private employee
d. Unemployed

10. How many years have you been in practice: _______________
11. Are you currently practicing i.e. do you attend to patients?
    a. Yes
    b. No

SECTION B- Knowledge

12. Do you know the occupations classified as 'Essential workers' by the Ministry of Health during the COVID-19 lockdown?
    a. Yes
    b. No
    c. Not sure
13. Which of the following professions do you consider as 'Essential workers'?
    a. Ophthalmology
    b. Optometry
    c. Ophthalmic nurse
    d. None of the above
    e. All of the above
14. How confident/informed do you feel in the Federal Ministry of Health (FMoH) guidelines that currently do not consider Eye care practitioners as 'Essential workers'?
    a. Extremely confident
    b. Very confident
    c. Somewhat confident
    d. Not so confident
    e. Not at all confident
15. Which of the following are recommended PPE by the NCDC in preventing COVID-19 transmission, during consultation of confirmed/suspected cases for health care workers? (Select all applicable)
    a. Hand hygiene
    b. Gloves
    c. Fluid resistant surgical face masks
    d. Eye protection
    e. Long sleeve disposable water resistant gown
    f. Ventilators
    g. Fluid proof apron
    h. Respiratory protection FFP2/N99
    i. None of the above
16. Have you had any training in PPE for the prevention of COVID-19
    a. Yes
    b. No
17. If yes, who provided the training?
    a. Employer
    b. Self
    c. Online
    d. Other (please specify): _____________

18. To what extent did the information about COVID-19 circulated by your professional body or Federal ministry of health equip you to confidently attend to your patients?
    a. Extremely useful
    b. Very useful
    c. Somewhat useful
    d. Not so useful
    e. Not at all useful

SECTION C- Practice during COVID-19

19. Do you feel at risk of COVID-19 infection as an eye care practitioner
    a. Yes
    b. No
    c. Not sure
20. During the COVID-19 lockdown, how confident do you feel attending to any patient?
    a. Extremely confident
    b. Very confident
    c. Somewhat confident
    d. Not so confident
    e. Not at all confident
21. How confident do you feel attending to a patient with or at risk of COVID-19?
    a. Extremely confident
    b. Very confident
    c. Somewhat confident
    d. Not so confident
    e. Not at all confident
22. After the lockdown, how confident would you feel attending to any patient?
    a. Extremely confident
    b. Very confident
    c. Somewhat confident
    d. Not so confident
    e. Not at all confident
23. How much would COVID-19 change the way you practice?
    a. Very much
    b. Moderately
    c. Very little
    d. Not at all
24. Did you attend to any patient during the COVID-19 lockdown?
    a. Yes
    b. No
25. If yes , what means did you attend to the patients (You can select more than one option)
    a. Physical consultation
    b. Physical consultation at patients preferred location
    c. Over the phone
    d. Video conference call e.g. Zoom, Skype or Telemedicine
    e. Social media
    f. Others (please specify): ____________________

26. What method did you adopt to disseminate information about COVID-19 to your patients? You can select more than one option
    a. Never did
    b. Emails
    c. Phone calls
    d. Social media
    e. Text message
    f. Website information
    g. Others (please specify): ________________
27. Which clinical procedures have you discontinued since the beginning of the COVID19 pandemic? You can select more than one option
    a. None
    b. Visual acuity
    c. Refraction
    d. Ophthalmoscopy
    e. Slit lamp examination
    f. Tonometry
    g. All
    h. Others (please specify): _____________

SECTION D- Impact

28. Have you been affected by COVID-19?
    a. Yes
    b. No
29. If yes, in what way have you been affected by COVID-19?
    a. Financial loss
    b. Lost job
    c. Pay cut
    d. Lost someone
    e. Contracted COVID19
30. Has any member of your family been affected by COVID 19
    a. Yes
    b. No
31. If yes, in what way have they been affected by COVID-19?
    a. Financial loss
    b. Lost job
    c. Pay cut
    d. Lost someone
    e. Contracted COVID19
32. Do you have fears of job insecurity due to the lockdown or COVID-19 pandemic?
    a. Yes
    b. No
    c. Not sure
33. Do you have health insurance cover for yourself/family or practice?
    a. Yes
    b. No

34. If no, considering the impact of COVID-19 pandemic, have you considered taking health insurance cover for yourself/family or practice?
    a. Yes
    b. No
    c. Not sure

SECTION E- Guidelines

35. Do you feel your professional regulatory board has provided sufficient guidance on identifying patients at risk or with COVID-19 during practice?
    a. Yes
    b. No
    c. Not sure
36. Has your professional association provided any guidance during the COVID-19 pandemic?
    a. Yes
    b. No
37. If yes, do you find these guidelines adequate and useful?
    a. Yes
    b. No

Thank you for participating.
